# A systematic method introduced a common lncRNA-miRNA-mRNA network in the different stages of prostate cancer

**DOI:** 10.3389/fonc.2023.1142275

**Published:** 2023-05-08

**Authors:** Gelareh Vahabzadeh, Solmaz Khalighfard, Ali Mohammad Alizadeh, Mahsa Yaghobinejad, Mahta Mardani, Tayebeh Rastegar, Mahmood Barati, Morad Roudbaraki, Ebrahim Esmati, Mohammad Babaei, Ali Kazemian

**Affiliations:** ^1^ Razi Drug Research Center, Iran University of Medical Sciences, Tehran, Iran; ^2^ Research Center on Developing Advanced Technologies, Tehran, Iran; ^3^ Cancer Research Center, Cancer Institute, Tehran University of Medical Sciences, Tehran, Iran; ^4^ Department of Anatomy, School of Medicine Tehran University of Medical Sciences, Tehran, Iran; ^5^ Department of Medical Biotechnology, Faculty of Allied Medicine, Iran University of Medical Sciences, Tehran, Iran; ^6^ Laboratory of Cell Physiology, Inserm U1003, University of Lille, Villeneuve d’Ascq, France; ^7^ Radiation Oncology Research Center, Cancer Institute, Tehran University of Medical Sciences, Tehran, Iran

**Keywords:** prostate cancer, biomarkers, Gleason, PSA, network

## Abstract

**Introduction:**

The present study aimed to investigate the interaction of the common lncRNA-miRNA-mRNA network involved in signaling pathways in different stages of prostate cancer (PCa) by using bioinformatics and experimental methods.

**Methods:**

Seventy subjects included sixty PCa patients in Local, Locally Advanced, Biochemical Relapse, Metastatic, and Benign stages, and ten healthy subjects were entered into the current study. The mRNAs with significant expression differences were first found using the GEO database. The candidate hub genes were then identified by analyzing Cytohubba and MCODE software. Cytoscape, GO Term, and KEGG software determined hub genes and critical pathways. The expression of candidate lncRNAs, miRNAs, and mRNAs was then assessed using Real-Time PCR and ELISA techniques.

**Results:**

4 lncRNAs, 5 miRNAs, and 15 common target genes were detected in PCa patients compared with the healthy group. Unlike the tumor suppressors, the expression levels of common onco-lncRNAs, oncomiRNAs, and oncogenes showed a considerable increase in patients with advanced stages; Biochemical Relapse and Metastatic, in comparison to the primary stages; Local and Locally Advanced. Additionally, their expression levels significantly increased with a higher Gleason score than a lower one.

**Conclusion:**

Identifying a common lncRNA-miRNA-mRNA network associated with prostate cancer may be clinically valuable as potential predictive biomarkers. They can also serve as novel therapeutic targets for PCa patients.

## 1 Introduction

Prostate cancer (PCa) is the second most diagnosed cancer in males worldwide and causes more than 350,000 deaths annually (1). Rectal examinations and prostate-specific antigen (PSA) levels are the first steps to check for PCa. PSA evaluation is beneficial for early diagnosis, but insufficient for disease grouping. Because a high value might be present in a person without cancer, and a low value can be present in someone with cancer, PSA values are difficult to interpret (2, 3). Prostate biopsies can also cause considerable infectious complications, psychological damage, and financial costs. So, the development of novel molecular biomarkers for PCa detection has allowed for better therapeutic target evaluations and prognostic assessments (4). Indeed, it is essential to explore molecular mechanisms, such as non-coding RNAs (lncRNA and miRNA), and identify novel targets for developing predictive, prognostic, and therapeutic goals for PCa.

Non-coding RNAs are specific, accurate, and accessible noninvasively, which makes them appealing for use as biomarkers for identifying tumor presence and subtypes. They control numerous biological processes, including cell proliferation, differentiation, and apoptosis (5). They are also recognized as critical regulators in various networks as both tumor repressors and oncogenic. In this case, lncRNA and miRNA interaction by integrating mRNA can provide opportunities for further experimental studies and improved biomarker predictions for developing novel diagnostic approaches. For example, miR-21 is increased in PCa (6, 7), and its overexpression can activate TGF-β and Hedgehog signaling pathways, promoting invasion and metastasis (8). Therefore, detecting non-coding RNAs, actively released from tumor cells into body fluids, makes them suitable as diagnostic and therapeutic biomarkers (9). Herein, we aim to investigate the interactions between common lncRNA-miRNA-mRNA networks in patients with prostate cancer. We hypothesize that a better understanding of the underlying mechanisms involved in this network and identifying commonly expressed biomarkers at different stages of PCa can lead to developing predictive, prognostic, and therapeutic goals.

## 2 Methods

### 2.1 The data collection of PCa datasets

Expression profiles of mRNAs (GSE16120, GSE119195, GSE62872, GSE30994, GSE69223, GSE55945, GSE68555, GSE51066, GSE134499, GSE36135, GSE3325, GSE116918, GSE70770, and GSE35988) from PCa with different tumor stages (local, locally-advanced, biochemical relapse, and metastatic), normal samples, and benign tumor samples were extracted from the Gene Expression Omnibus (GEO) (https://www.ncbi.nlm.nih.gov/geo/) database. Gene expression information for Agilent-012097 Human 1A Microarray, Affymetrix Human Gene 1.0 ST Array, Agilent-014850 Whole Human Genome Microarray, Affymetrix Human Genome U133 Plus 2.0 Array, Affymetrix Human Genome U95A Array, Affymetrix Genome-Wide Human SNP 6.0 Array, Almac Diagnostics Prostate Disease-Specific Array, Agilent-039494 SurePrint G3 Human Gene Expression v2 8x60K Microarray, and Agilent-014698 Human Genome CGH Microarray 105A were obtained from prostate cancer patients with different tumor stages. GEPIA2 and cBioPortal analyze differentially expressed genes (DEGs) from the TCGA (10). The expression profile pattern of PCa patients was compared to that of healthy individuals to identify DEGs. The GSEs’ data were downloaded using the GEOquery R package. We analyzed the selected lncRNAs, miRNAs, and genes with P-value < 0.05 and |LogFC| > 1 in the datasets as differentially expressed genes (DEGs), differentially expressed miRNAs (DEMs), and differentially expressed lncRNAs (DELs) (11).

### 2.2 Identification of lncRNAs and miRNAs of candidate target genes

**Figure 1:**
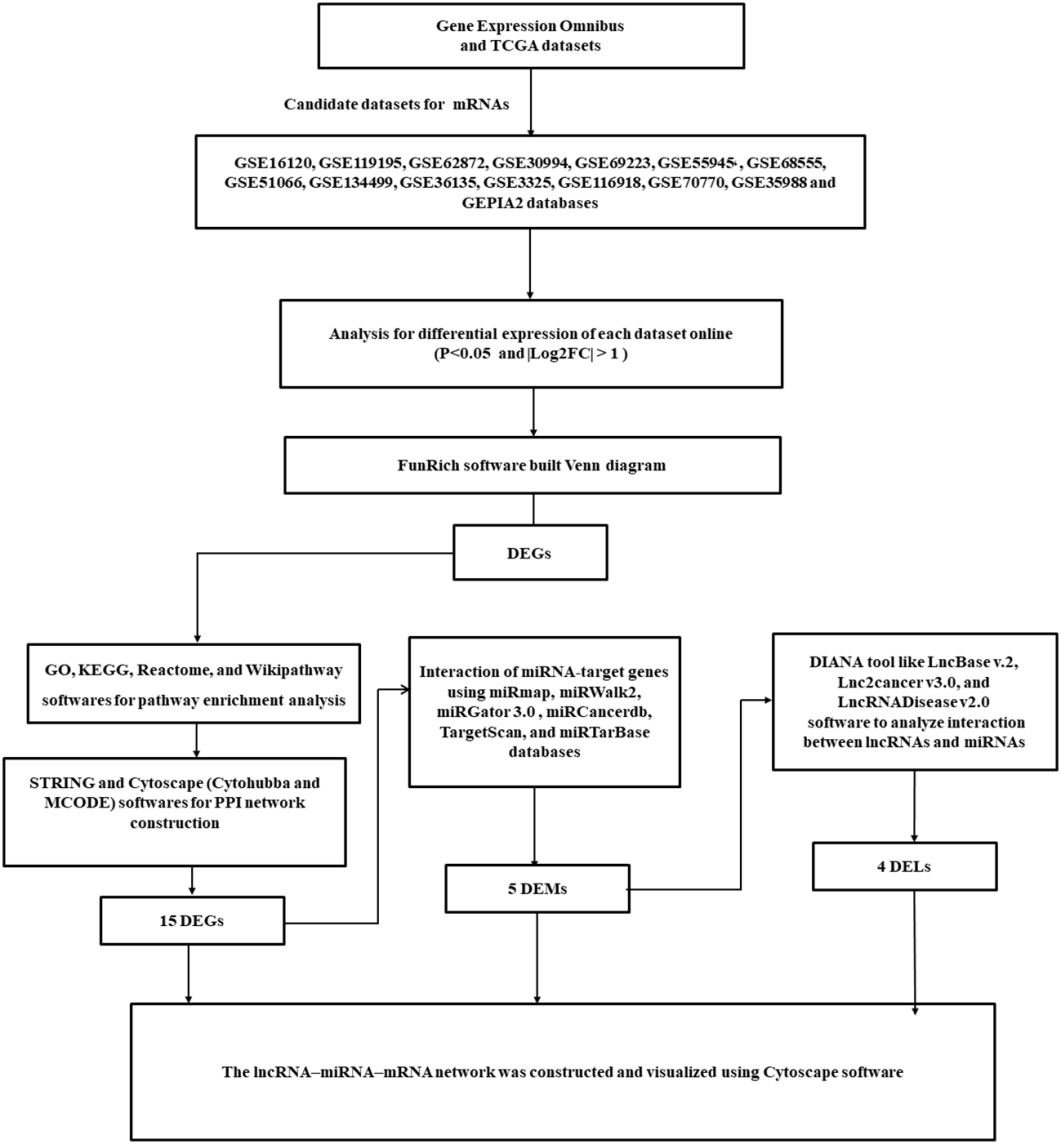
A flowchart diagram for analyzing bioinformatics data.

MiRNAs of candidate genes were identified in the miRWalk2, miRmap, OncomiR, miRGator 3.0, and miRCancerdb databases of the TCGA dataset (10). Moreover, LncRNA2target, TANRIC, LncRNADisease, Lnc2Cancer v3.0, and LncBase of the TCGA dataset were collected to identify candidate lncRNAs (11). **Figure 1** shows the bioinformatics analyses.

### 2.3 Enrichment analysis for DEGs

Cytoscape software uses the ClueGo tool to identify gene ontologies (11). It is accepted that p < 0.05 is statistically significant in GO analysis. Moreover, the Reactome and WikiPathway databases were used to identify signaling pathways (10). The Enrichr software performed pathway enrichment analyses of the DEGs.

### 2.4 An analysis of DEGs by protein-protein interaction (PPI)

STRING software predicted PPI network information. Candidate hub genes were identified by Cytohubba (Degree = 15) and MCODE (Degree Cutoff = 2, Node score cutoff = 2, and K-score = 2) tools on Cytoscape software. A ceRNA network between lncRNAs, miRNAs, and mRNAs was constructed using Cytoscape 3.7.1 software (10, 11).

### 2.5 Experimental design and sampling

Eighty PCa patients and ten healthy subjects were entered into the current study from September 2019 to September 2020. The eligible cases were categorized into six groups: Local (L, N = 22); those whose tumor has not spread outside the prostate capsule, Locally Advanced (LA, N = 9); those whose tumor has invaded the seminal vesicles and other pelvic organs, Biochemical Relapse (BR, N = 11); those in which PSA increased again after the treatment period, Metastatic (MET, N = 8); those whose cancer tissue has spread in secondary areas such as lungs and bones, and Benign Prostate Hypertrophy (BPH, N = 10) patients, and healthy samples based on pathologic reports.

#### 2.5.1 Inclusion criteria for PCa patients

1. Voluntary participation
2. Age 50−80 years
3. PCa patients (L, LA, BR, MET, and BPH)
4. Gleason degree 3-10
5. No family history of cancer

#### 2.5.2 Inclusion criteria for healthy subjects

1. Voluntary participation
2. Age 50−80 years
3. No family history of cancer
4. Normal PSA

#### 2.5.3 Exclusion criteria for PCa patients

1. A history of other cancers
2. A history of diabetes
3. A hypertension history
4. Autoimmune diseases

### 2.6 Real-time PCR analysis

Approximately 10 mL of blood samples were taken from all participants and centrifuged. The total RNA was then extracted by adding 1 mL TRIzol (Beijing Tian-166 Gen Biotech Co., Ltd.) to each sample according to the manufacturer’s instructions. The miRcute miRNA First-Strand cDNA Synthesis kit (Beijing Tiangen Biotech Co., Ltd.) and the cDNA Synthesis Kit Manual (TAKARA BIO INC. Cat. 6 30 v.0708) were utilized. The miRcute miRNA qPCR Detection Kit (Tiangen Biotech Co., Ltd.) was used for the qRT-PCR assays, and the SYBR Green method (AccuPower Green Star qPCR Master Mix; Bioneer, Korea) was used for mRNAs and lncRNAs using the ABI StepOne Plus System (Applied Biosystems; Thermo Fisher Scientific, Inc.) (10). For normalization, U6 and B-actin were used as internal controls. The fold changes of candidate lncRNAs, miRNAs, and mRNAs were calculated by the equation −ΔCT (11). The **Supplementary File (S1)** shows the primers’ list.

### 2.7 Statistical tests

The statistical tests were conducted with GraphPad Prism 7.04 (San Diego, CA). The K-S test estimates data normality. ANOVA and t-tests were applied to analyze multiple and two groups. The Mann-Whitney U test evaluated nonparametric data. REST analyzed the PCR data. Regression analysis was performed to avoid bias in confounding variables. P < 0.05 was considered as a level of significance.

## 3 Results

### 3.1 Identification of hub genes at different stages of prostate cancer

**Figure 2:**
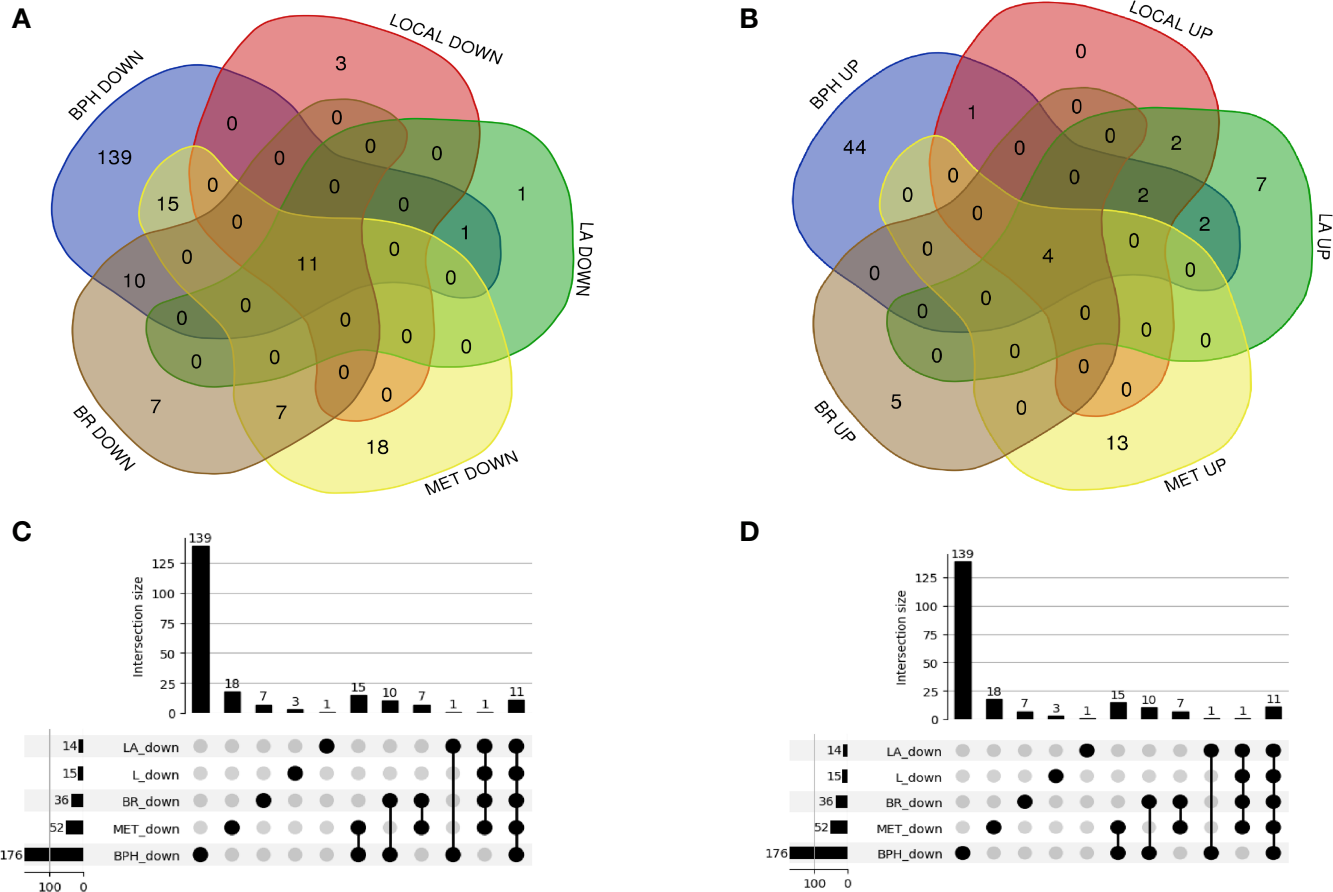
A Venn diagram and UpSet plot of the differently expressed mRNAs between the GEO and TCGA datasets **(A, B)** 23 genes (9 up-regulation and 14 down-regulation) in the local group, 30 genes (17 up-regulation and 13 down-regulation) in the locally advanced group, 68 genes (17 up-regulation and 51 down-regulation) in the metastatic group, 44 genes (9 up-regulation and 35 down-regulation) in the biochemical relapse group, and 228 genes (53 up-regulation and 175 down-regulation) in the BPH group were differentially expressed compared to healthy samples. **(C, D)** the UpSet plot of overlapped differential expression genes among candidate groups.

A total of 23 genes (9 up-regulation and 14 down-regulation) in the local group, 30 genes (17 up-regulation and 13 down-regulation) in the locally-advanced group, 68 genes (17 up-regulation and 51 down-regulation) in the metastatic group, 44 genes (9 up-regulation and 35 down-regulation) in the biochemical relapse group, and 228 genes (53 up-regulation and 175 down-regulation) in the BPH group (**Figures 2A, B**) were analyzed from GEO and TCGA datasets to identify differentially expressed genes in prostate cancer and healthy samples (**Supplementary S2**). A Venn diagram and Upset plot of DEGs at different stages of the disease were drawn by calculating and drawing custom Venn diagrams using the online tools and FunRich_3.1.3 software and upset plot using Up Set R package (**Figure 2**). Furthermore, differentially expressed miRNAs (**Table 1**) and lncRNAs (**Table 2**) were determined at various stages of prostate cancer.

**Table 1:** Interaction between candidate mRNAs and target miRNAs in PCa.

Hub genes	Targeting miRNAs
ALB	miRNA-195
CD44	miRNA-200c, miRNA-149, miRNA-182
CXCL12	miRNA-149, miRNA-491, miRNA-182
IL10	miRNA-149, miRNA-491
ITGB3	miRNA-149, miRNA-195
BDNF	miRNA-149, miRNA-200c, miRNA-49, miRNA-195, miRNA-182
MET	miRNA-200c
PLG	miRNA-149
APOE	miRNA-149
MMP1	miRNA-149
F2	miRNA-182
ITGA6	miRNA-195, miRNA-149
ITGA5	miRNA-149, miRNA-182, miRNA-200c
FGF18	miRNA-195
FAP	miRNA-491

**Table 2:** Interaction between candidate miRNAs and target lncRNAs in PCa.

LncRNAs	Targeting miRNAs
NEAT1	miRNA-491
MALAT1	miRNA-182
PCAT19	miRNA-195, miRNA-200c, miRNA-182
CASC2	miRNA-491

### 3.2 Identified hub genes by GO term

**Figure 3:**
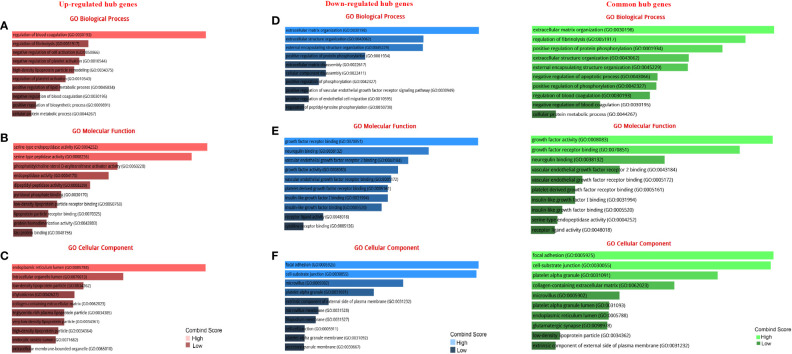
GO terms of DEGs between up- and down-regulated hub genes. **(A)** GO analysis of up-regulated DEGs related to biological processes. **(B)** GO analysis of up-regulated DEGs related to molecular functions. **(C)** GO analysis of up-regulated DEGs linked to cellular components. **(D)** GO analysis of down-regulated DEGs related to biological processes. **(E)** GO analysis of down-regulated DEGs linked to molecular functions. **(F)** GO analysis of down-regulated DEGs linked to cellular components.

**Figure 4:**
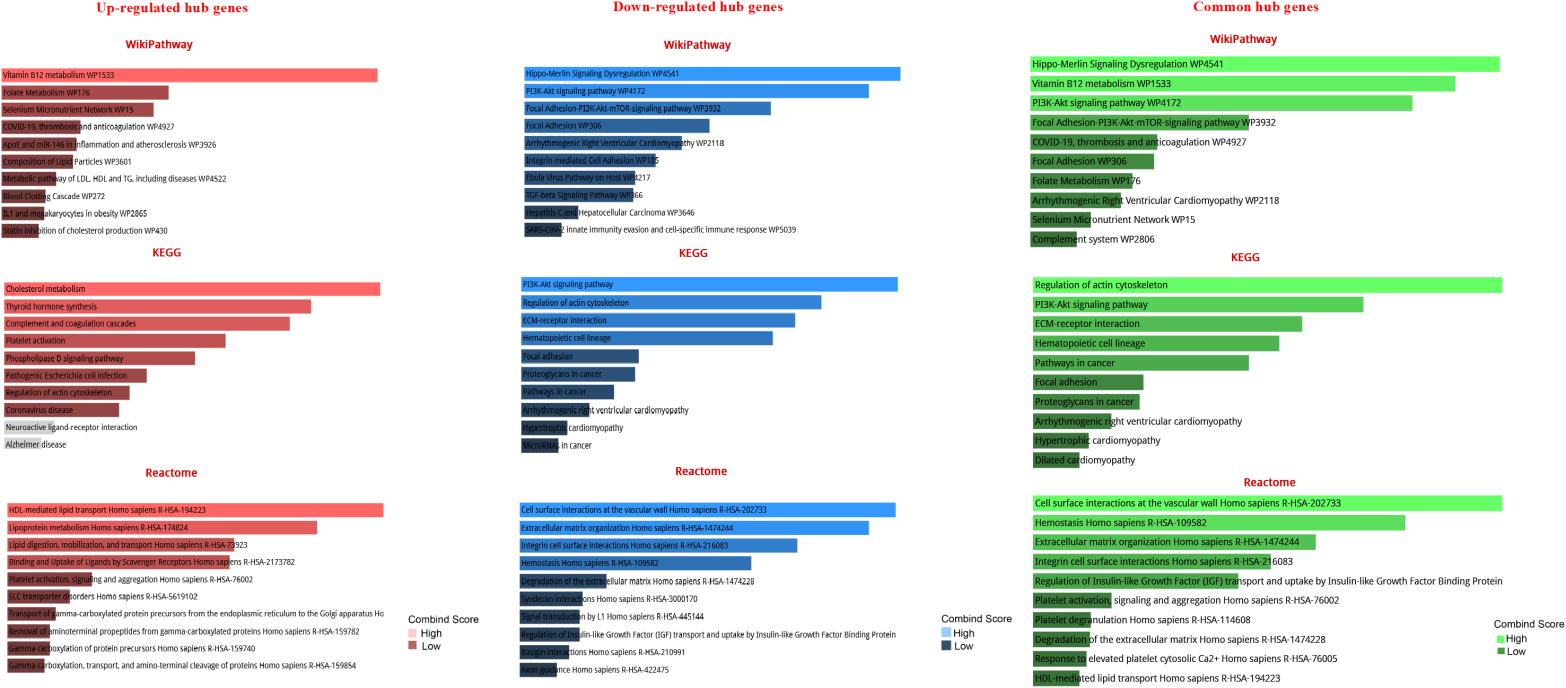
Pathway-based enrichment analysis of high-expressed hub genes and low-expressed hub genes at different stages of prostate cancer The top 10 functional pathways were associated with DEGs through WikiPathway, Reactome, and KEGG analyses with a p-value of less than 0.05.

The GO analysis revealed the enriched pathways identified in the DEGs (**Figure 3**). Moreover, the signaling pathways involved in the hub genes from the Reactome, KEGG, and Wikipathway databases were listed in **Figure 4**. Databases’ common pathways involve focal adhesion and cell proliferation, such as PI3K/AKT/mTOR (**Figure 4**).

### 3.3 Validation of hub genes by PPI network

**Figure 5:**
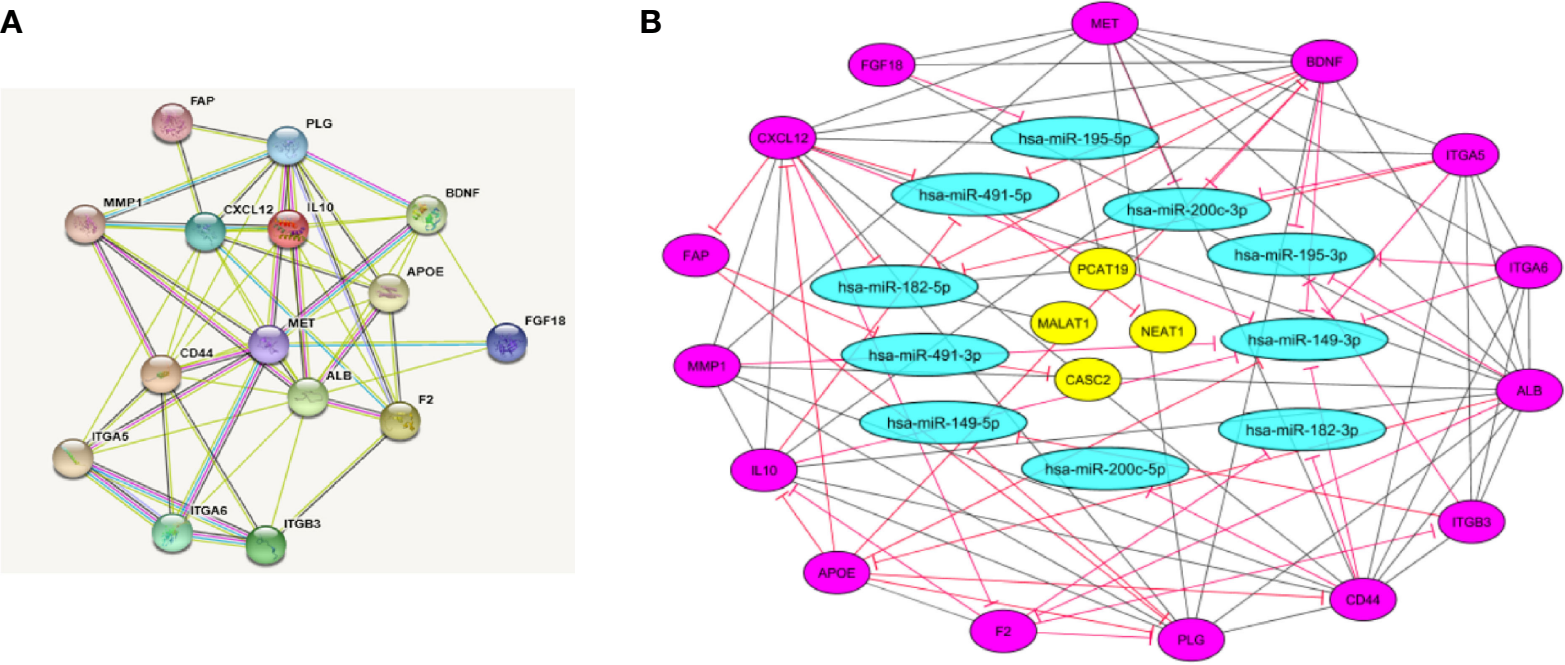
Protein-protein interaction (PPI) network construction. **(A)** PPI network was constructed with DEGs from GEO and TCGA datasets that show the interaction between hub genes by STRING software. **(B)** LncRNA-miRNA-mRNA network by Cytoscape software. The network includes 29 nodes and 88 edges. The yellow ellipse, blue ellipse, and purple ellipse oval represent lncRNAs, miRNAs, and mRNAs, respectively.

**Figure 6:**
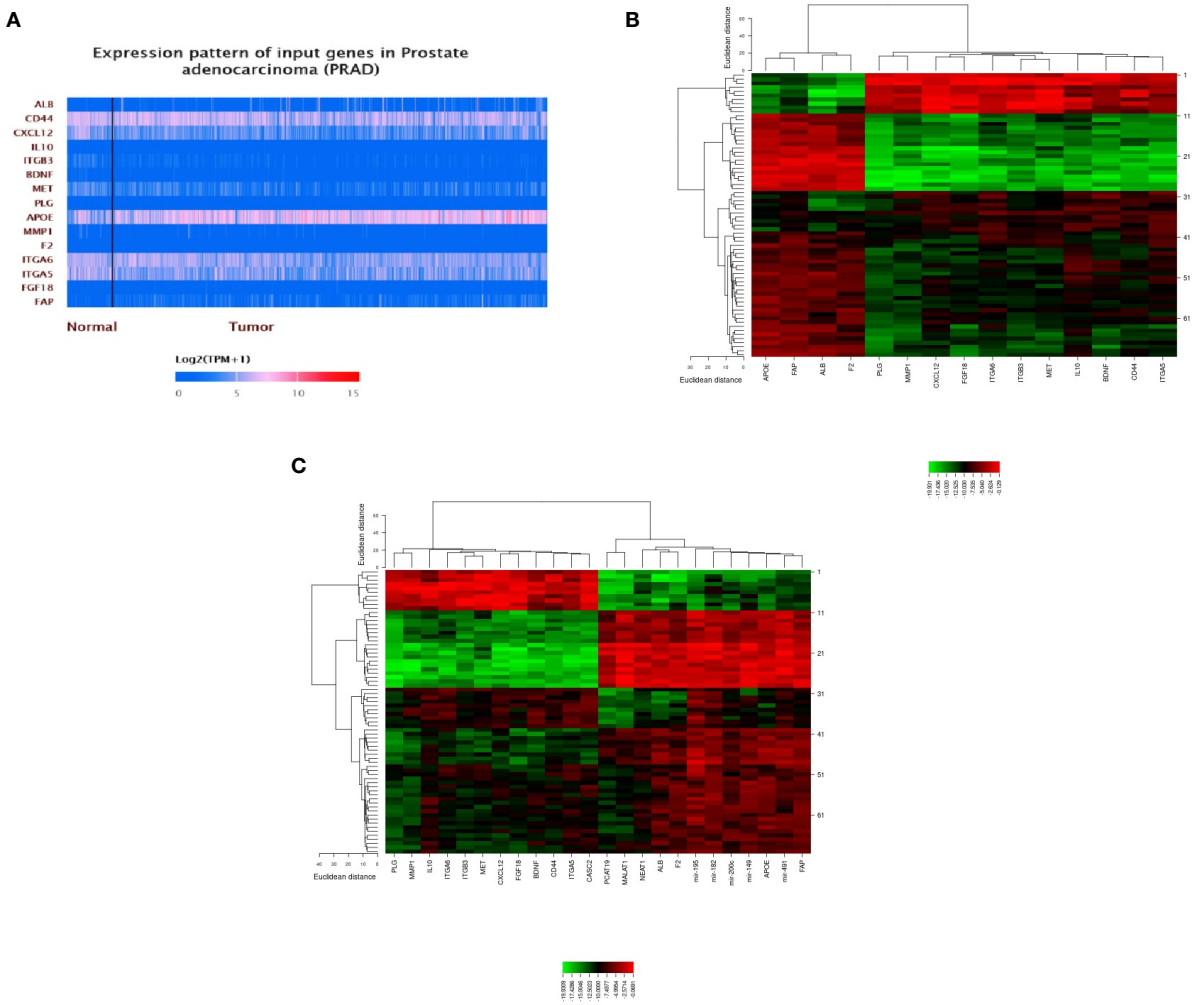
A plot heatmap showing the gene expression profile of DEGs in both bioinformatics **(A)** and experiment data (-ΔCT) **(B, C)** in PCa patients. The green color indicates down-regulated genes, and the red color indicates up-regulated genes between tumor and healthy samples.

STRING software was used to construct and predict PPI network information (**Figure 5A**). Candidate hub genes were identified by analyzing Cytohubba and MCODE software (**Figure 5B**). Our results showed 4 lncRNAs, 5 miRNAs, and 15 mRNAs from a regulatory lncRNA-miRNA-mRNA network in PCa patients (**Figure 5B**). Besides, a heatmap map of candidate lncRNAs’, miRNAs’, and mRNAs’ expressions was drawn by CIMminer software (**Figure 6**).

### 3.4 Patients’ characteristics

The clinicopathological characteristics of the participants are summarized in **Table 3**. There were no significant differences in age, BMI, and prostate volume in the different groups. The most considerable tumor volume (cm^3^) belonged to the metastatic group (58.3 ± 7.7), and most prostate cancer patients had adenocarcinoma in pathology assessment. Except for the biochemical relapse group (due to surgery and complete prostate resection), serum PSA levels increased significantly with disease progression in other groups. All patients in the biochemical relapse group were given hormone therapy and a radical prostatectomy. All metastatic patients received hormone therapy and palliative radiotherapy. In the other groups, most received hormone therapy alone.

**Table 3:** Demographic data of PCa patients. .

Groups Index	Healthy	BPH	L	LA	BR	MET
Age (year)	60 ± 7	62 ± 6	59 ± 8	61 ± 7	58 ± 6	63 ± 5
BMI	28.2 ± 1.8	27 ± 2.0	26.2 ± 1.9	25.7 ± 3.1	23.2 ± 1.2	23.1 ± 1.3
Prostate volume (cm^3^)	50.2 ± 3.5	54.5 ± 5.5	52.3 ± 3.4	49.7 ± 4.4	–	51.4 ± 2.5
Tumor size (cm^3^)	–	–	40.3 ± 3.2	46.9 ± 4.6	–	58.3 ± 7.7
Pathology (%)						
Adenocarcinoma Adeno + DC Adeno+ SCNEC	- - -	- - -	92.8 7.2 -	86.8 10.8 3.6	82 10.8 7.2	71.2 18 10.8
PSA (ng/ml)	1.5 ± 0.4	4.5 ± 2.3	13.2 ± 10.2	45.9 ± 14.3	0.5 ± 0.1	59.5 ± 14.6
Gleason Score (%)						
6 7 8 9 10	- - - - -	89.2 10.8 - - -	74.8 18 7.2 - -	3.6 32.4 64 - -	- 3.6 14.4 43.2 38.8	- - 3.6 32.4 64
Clinical T stage (%)						
2a 2b 2c 3a 3b 3c	- - - - - -	- - - - - -	64.8 35.2 - - - -	31.6 61.2 7.2 - - -	- - 7.2 14.4 31.6 46.8	- - 7.2 10.8 35.2 46.8
Treatment (%)						
ADT alone ADT + ECTOMY ADT + RT	- - -	- - -	64.8 35.2 -	82 - 18	- 100 -	- - 100

Data were calculated based on mean ± SD and percentage.

BPH, Benign Prostatic Hyperplasia; L, Local; LA, Locally Advanced; BR, Biochemical Relapse; MET, Metastatic; BMI, Body mass index; ADT, Androgen deprivation therapy; RT, Radiation therapy; SCNEC, Small cell neuroendocrine carcinoma.

### 3.5 The correlation of clinicopathological status with commonly expressed biomarkers

The expression level of onco-lncRNAs, including PCAT19, MALAT1, and NEAT1, and the tumor suppressor lncRNA, CASC2, was increased and decreased, respectively, in PCa patients compared to the healthy group (P < 0.05) (**Table 4**). Interestingly, the altered expression levels of lncRNAs were significantly associated with increased tumor stages (**Table 4**) and Gleason score (**Table 5**).

**Table 4:** The expression levels of candidate lncRNAs at different stages of PCa patients.

Groups LncRNAs	Healthy	BPH	L	LA	BR	MET
Onco-lncRNAs						
MALAT1	-17.5 ± 0.9	**-**16.5 ± 0.9	**-**10.5 ± 1.2^*^	**-**9.7 ± 0.9^*^	**-**2.1 ± 0.7 ^*,#^	**-**3.5 ± 1.3 ^*,#^
PCAT19	-17.2 ± 1.2	**-**14.4 ± 1.5	**-**11.3 ± 0.1^*^	**-**8.4 ± 1.1^*^	**-**5.1 ± 1.0 ^*,#^	**-**4.6 ± 0.7 ^*,#^
NEAT1	-13.8 ± 1.7	-13.0 ± 1.2	**-**9.2± 1.2^*^	**-**7.4 ± 0.9^*^	**-**2.3± 1.0 ^*,#^	**-**1.9 ± 1.3 ^*,#^
Tumor suppressor lncRNAs						
CASC2	-1.5 ± 0.7	**-**1.7 ± 0.8	**-**8.9 ± 1.2^*^	**-**12.2 ± 1.2^*^	**-**17.8 ± 0.9 ^*,#^	**-**18.0 ± 0.8 ^*,#^

Data were calculated based on mean ± SD and –ΔCT.

^*^P <0.05 compared to the Healthy and BPH groups.

^#^P <0.05 compared to the L and LA groups.

BPH, Benign Prostatic Hyperplasia; L, Local; LA, Locally Advanced; BR, Biochemical Relapse; MET, Metastatic.

**Table 5:** The expression levels of candidate lncRNAs with Gleason degree in PCa patients.

Groups LncRNAs	Health	GS ≤ 6	GS 8-10
Onco-lncRNAs			
MALAT1	-17.5 ± 0.9	**-**10.3 ± 1.2^*^	**-**2.7 ± 1.2 ^#^
PCAT19	-17.2 ± 1.2	**-**10.4 ± 1.7 ^*^	**-**4.9 ± 0.9 ^#^
NEAT1	-13.8 ± 1.7	**-**8.7 ± 1.4 ^*^	**-**2.1 ± 1.1 ^#^
Tumor suppressor lncRNAs			
CASC2	-1.5 ± 0.7	**-**9.8 ± 1.9 ^*^	**-**17.9 ± 0.9 ^#^

Data were calculated based on mean ± SD and -ΔCT. GS, Gleason score.

^*^P <0.05 compared to the Healthy group.

^#^P <0.05 compared to the GS ≤ 6.

Except for miRNA-195, the expression levels of the oncomiRs, including miRNA-491, miRNA-182, miRNA-149, and miRNA-200c, showed a significant increase in PCa patients compared to the healthy group (P < 0.05) (**Table 6**). Interestingly, except for miRNA-195, the expression levels of the mentioned other miRNAs were significantly associated with increased tumor stages (**Table 6**) and Gleason score (**Table 7**).

**Table 6:** The expression levels of candidate miRNAs in different stages of PCa patients.

Groups miRNAs	Health	BPH	L	LA	BR	MET
miRNA-195	-15.2 ± 1.5	-14.8 ± 2.5	**-**6.9 ± 0.9^*^	**-**5.0 ± 1.0 ^*^	-1.8 ± 0.9 ^*,#^	**-**1.4 ± 0.8^*,#^
miRNA-149	-13 ± 2.7	-12.0 ± 1.4	**-**6.2 ± 1.2^*^	**-**5.4 ± 0.8 ^*^	-2.2 ± 0.8 ^*,#^	**-**1.8 ± 1.1^*,#^
miRNA-182	-11.9 ± 2.5	-10.0 ± 1.3	**-**5.8 ± 1.0^*^	**-**4.6 ± 1.0 ^*^	-1.9 ± 1.3 ^*,#^	**-**1.2 ± 1.1^*,#^
miRNA-491	-12.4 ± 1.8	-11.3 ± 1.4	**-**6.9 ± 1.1^*^	**-**4.6 ± 1.1 ^*^	-1.5 ± 1.1 ^*,#^	**-**1.3 ± 0.9^*,#^
miRNA-200c	-13.8 ± 2	-12.0 ± 1.8	**-**7.2 ± 1.2^*^	-6.7 ± 0.9 ^*^	-2.7 ± 0.9^*,#^	**-**1.7 ± 1.1^*,#^

Data were calculated based on mean ± SD and –ΔCT.

^*^P <0.05 compared to the Health and BPH groups.

^#^P <0.05 compared to the L and LA groups.

BPH, Benign Prostatic Hyperplasia; L, Local; LA, Locally Advanced; BR, Biochemical Relapse; MET, Metastatic.

**Table 7:** The expression levels of candidate miRNAs with Gleason degree in PCa patients.

Groups miRNAs	Healthy	GS ≤ 6	GS 8-10
miRNA-195	-15.2 ± 1.5	**-**6.3 ± 1.2 ^*^	-1.7 ± 0.9 **^#^**
miRNA-149	-13 ± 2.7	**-**5.9 ± 1.2 ^*^	-2.0 ± 0.9 **^#^**
miRNA-182	-11.9 ± 2.5	**-**5.4 ± 1.1 ^*^	-1.6 ± 1.2 **^#^**
miRNA-491	-12.4 ± 1.8	**-**6.2 ± 1.5 ^*^	-1.4 ± 1.0 **^#^**
miRNA-200c	-13.8 ± 2	**-**7.0 ± 1.2 ^*^	-2.2 ± 1.1 **^#^**

Data were calculated based on mean ± SD and -ΔCT. GS, Gleason score.

^*^P <0.05 compared to the healthy group.

^#^P <0.05 compared to the GS ≤ 6 group.

The expression levels of the oncogenes, including ALB, APOE, F2, and FAP, and the tumor suppressor genes, including BDNF, MET, PLG, MMP1, ITGA6, ITGA5, FGF18, CD44, CXCL12, IL10, and ITGB3, were significantly increased and decreased, respectively, in PCa patients compared to the healthy group (P < 0.05) (**Table 8**). A significant correlation was also found between altered expression levels and increased tumor stages (**Table 8**) and Gleason scores (**Table 9**).

**Table 8:** The expression levels of candidate mRNAs in different stages of PCa patients.

Groups Genes	Healthy	BPH	L	LA	BR	MET
Oncogenes						
ALB	-16.3 ± 2.2	**-**14.0 ± 1.0	**-**7.3 ± 1.5 ^*^	**-**7.0 ± 1.4 ^*^	**-**3.0 ± 0.6 ^*,#^	**-**2.8 ± 0.6 ^*,#^
APOE	-14.5 ± 1.6	**-**13.0 ± 1.4	**-**6.3 ± 1.3 ^*^	**-**5.7 ± 0.9 ^*^	**-**2.1 ± 0.9 ^*,#^	**-**1.8 ± 1.0 ^*,#^
F2	-15.5 ± 2.8	**-**13.5 ± 4.0	**-**6.9 ± 1.2 ^*^	**-**6.3 ± 0.9 ^*^	**-**2.2 ± 0.7 ^*,#^	**-**1.0 ± 0.7 ^*,#^
FAP	-12.3 ± 1.0	**-**11.1 ± 1.9	**-**6.1 ± 0.7 ^*^	**-**5.0 ± 0.6 ^*^	**-**2.1 ± 0.8 ^*,#^	**-**1.9 ± 1.1 ^*,#^
Tumor suppressor genes						
CD44	-3.9 ± 2.3	**-**4.7 ± 1.5	**-**11.0 ± 1.5 ^*^	**-**11.1 ± 1.1 ^*^	**-**17.0 ± 0.7 ^*,#^	**-**17.8 ± 1.0 ^*,#^
CXCL12	-1.2 ± 0.7	**-**1.7 ± 1.8	**-**10.4 ± 1.5 ^*^	**-**11.1 ± 1.4 ^*^	**-**17.6 ± 1.7 ^*,#^	**-**17.9 ± 1.3 ^*,#^
IL10	-3.3 ± 2.5	**-**3.9 ± 1.6	**-**8.8 ± 1.4 ^*^	**-**9.5 ± 1.2 ^*^	**-**16.4 ± 1.3 ^*,#^	**-**17.2 ± 2.0 ^*,#^
ITGB3	-1.9 ± 1.4	**-**1.9 ± 0.4	**-**10.8 ± 1.3 ^*^	**-**11.2 ± 1.1 ^*^	**-**17.3 ± 1.5 ^*,#^	**-**18.1 ± 1.6 ^*,#^
BDNF	-3.8 ± 1.9	**-**3.9 ± 1.6	**-**9.9 ± 1.4 ^*^	**-**10.7 ± 1.3 ^*^	**-**17.0 ± 1.4 ^*,#^	**-**18.1 ± 1.9 ^*,#^
MET	-1.3 ± 1.2	**-**2.1 ± 1.2	**-**10.4 ± 1.5 ^*^	**-**10.8 ± 0.9 ^*^	**-**17.0 ± 1.7 ^*,#^	**-**18.3 ± 1.6 ^*,#^
PLG	-2.7 ± 1.2	**-**2.0 ± 0.3	**-**10.9 ± 0.8 ^*^	**-**11.0 ± 1.2 ^*^	**-**18.3 ± 0.9 ^*,#^	**-**19.1 ± 1.1 ^*,#^
MMP1	-1.9 ± 1.2	**-**2.1 ± 0.5	**-**11.9 ± 1.3 ^*^	**-**12.9 ± 1.2 ^*^	**-**17.5 ± 1.9 ^*,#^	**-**18.4 ± 2.4 ^*,#^
ITGA6	-2.1 ± 1.1	**-**1.7 ± 0.7	**-**9.8 ± 1.4 ^*^	**-**9.4 ± 1.1 ^*^	**-**16.7 ± 1.6 ^*,#^	**-**18.8 ± 1.7 ^*,#^
ITGA5	-3.9 ± 1.1	**-**2.1 ± 1.5	**-**8.8 ± 1.3 ^*^	**-**9.8 ± 0.7 ^*^	**-**17.4 ± 1.0 ^*,#^	**-**19.1 ± 1.6 ^*,#^
FGF18	-1.5 ± 1.1	**-**1.9 ± 0.5	**-**10.8 ± 1.1 ^*^	**-**11.3 ± 1.1 ^*^	**-**17.9 ± 1.2 ^*,#^	**-**19.0 ± 1.9 ^*,#^

Data were calculated based on mean ± SD and –ΔCT.

^*^P <0.05 compared to the Healthy and BPH groups. # P <0.05 compared to the L and LA groups.

BPH, Benign Prostatic Hyperplasia; L, Local; LA, Locally Advanced; BR, Biochemical Relapse; MET, Metastatic.

**Table 9:** The expression levels of candidate mRNAs with Gleason degree in PCa patients.

Groups Genes	Healthy	GS ≤ 6	GS 8-10
Oncogenes			
ALB	-16.3 ± 2.2	-7.2 ± 1.4 ^*^	**-**2.9 ± 0.6 **^#^**
APOE	-14.5 ± 1.6	**-**6.1 ± 1.2 ^*^	**-**2.0 ± 0.9 **^#^**
F2	-15.5 ± 2.8	**-**6.7 ± 1.1 ^*^	**-**1.7 ± 0.9 **^#^**
FAP	-12.3 ± 1.0	**-**5.8 ± 0.8 ^*^	**-**2.0 ± 0.9 **^#^**
Tumor suppressor genes			
CD44	-3.9 ± 2.3	**-**11.1 ± 1.3 ^*^	**-**17.3 ± 0.9 **^#^**
CXCL12	-1.2 ± 0.7	**-**10.6 ± 1.4 ^*^	**-**17.7 ± 1.5 **^#^**
IL10	-3.3 ± 2.5	**-**9.0 ± 1.4 ^*^	**-**16.7 ± 1.6 **^#^**
ITGB3	-1.9 ± 1.4	**-**10.9 ± 1.2 ^*^	**-**17.6 ± 1.5 **^#^**
BDNF	-3.8 ± 1.9	**-**10.1 ± 1.4 ^*^	**-**17.5 ± 1.7 **^#^**
MET	-1.3 ± 1.2	**-**10.5 ± 1.3 ^*^	**-**17.6 ± 1.7 **^#^**
PLG	-2.7 ± 1.2	**-**10.9 ± 0.9 ^*^	**-**18.6 ± 1.1 **^#^**
MMP1	-1.9 ± 1.2	**-**12.2 ± 1.3 ^*^	**-**17.9 ± 2.1 **^#^**
ITGA6	-2.1 ± 1.1	**-**9.7 ± 1.3 ^*^	**-**17.6 ± 1.9 **^#^**
ITGA5	-3.9 ± 1.1	**-**9.1 ± 1.3 ^*^	**-**18.1 ± 1.5 **^#^**
FGF18	-1.5 ± 1.1	**-**10.9 ± 1.1 ^*^	**-**18.4 ± 1.6 **^#^**

Data were calculated based on mean ± SD and -ΔCT. GS, Gleason score.

^*^P <0.05 compared to the Healthy group, # P <0.05 compared to the GS ≤ 6 group.

**Figure 7:**
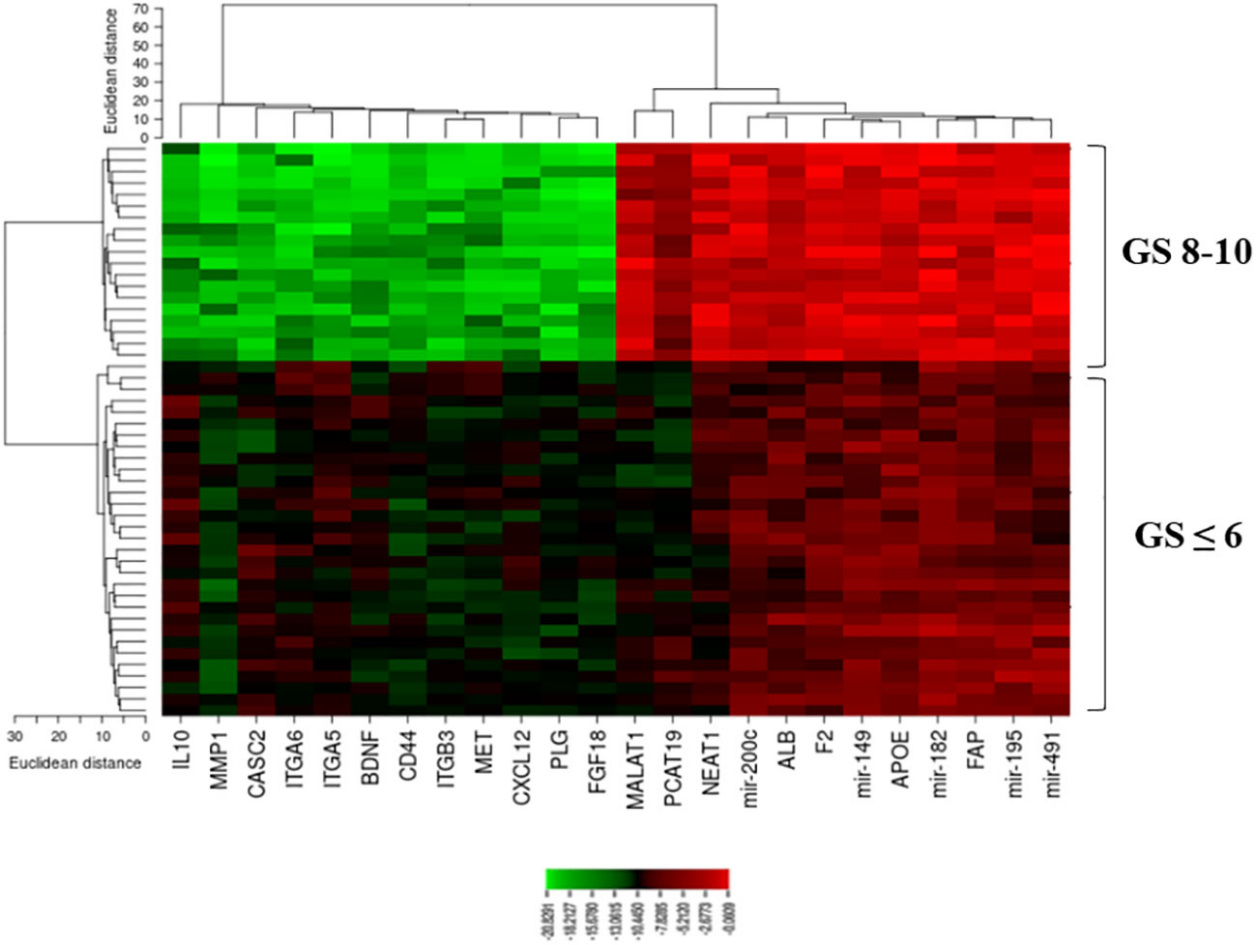
A plot heatmap showing the lncRNA-miRNA-mRNA network with Gleason score (≤ 6 and 8-10) in PCa patients. The green color indicates down-regulated lncRNA-miRNA-mRNA and the red color indicates up-regulated lncRNA-miRNA-mRNA.

A heatmap of the expression of candidate lncRNAs, miRNAs, and mRNAs with clinicopathological Gleason scores was also generated by CIMminer. We showed that the high expression of the onco-lncRNAs, oncomiRNAs, and oncogenes, and the low expression of the tumor suppressors have led to increases in Gleason scores (**Figure 7**).

Moreover, we compared candidate biomarkers’ profiles according to age. Our analysis showed no significant difference between lncRNA, miRNA, and mRNA expression with participants’ age.

## 4 Discussion

The present study aimed to investigate the underlying pathophysiological pathways involved in different stages of prostate cancer tumors. Our results revealed a significant reduction in tumor suppressor lncRNAs, miRNAs, and mRNAs alongside a considerable increase in onco-lncRNAs, oncomiRNAs, and oncogenes at different stages of prostate cancer. Likewise, these biomarkers with a higher Gleason score were significantly increased compared to tumors with lower Gleason scores. Furthermore, increased PSA levels were associated with advanced disease stages in prostate cancer. Thus, the interactions between lncRNAs, miRNAs, and mRNAs could contribute to finding novel biomarkers for assessing treatment response in PCa patients. Therefore, a better understanding of miRNA-lncRNA-mRNA signaling pathways may identify novel therapeutic targets for PCa patients.

Many patients suffering from advanced PCa will ultimately relapse after initial chemotherapy and hormone therapy-based regimens (12). Numerous genetic alterations concerning cell death and survival signaling processes may be responsible for PCa induction in this setting. Over the past decade, several investigations have been conducted concerning prostate cancer-related protein-encoding genes (13). However, only 2% of human transcriptomes are protein-coding RNAs (14). In order to better understand the underlying mechanisms of PCa initiation and progression, non-coding RNAs should be investigated (15, 16). Previous studies demonstrated that ceRNAs play pivotal roles in Pca progression and metastasis and even resistance to treatment (17). Recently, Liu et al. (18) showed that some miRNAs, such as miRNA-141, could reduce cancer growth and metastasis through several mechanisms (18). They also reported that miRNA-34a could directly prevent Pca regeneration and metastasis by suppressing the CD44 gene (19). Scheffer et al. (20) indicated that miRNAs -96, -149, -181b, -182, -205, and -375 were differentially expressed in prostate tumor tissues, while miRNAs -31, -96, and -205 exhibited a significant correlation with Gleason score (20). Furthermore, Borkowetz et al. (21) revealed that PSA’s diagnostic potential will significantly increase in combination with miRNA-16 and miRNA-195 as diagnostic biomarkers (21). Similarly, our results showed that the expression levels of miRNAs -491, -182, -149, and -200c were significantly increased in PCa patients compared to the healthy group which was significantly associated with advanced tumor stages.

Moreover, Aiello et al. (22) demonstrated that lncRNAs, such as HOTAIR and MALAT1, were associated with both ERα/ERβ steroid receptors in prostate cancer patients. Inhibition of MALAT1 led to better treatment response (22). Another study indicated that MALAT1 overexpression was associated with poor prognostic markers, including high Gleason score, metastasis stage, and serum PSA > 20 ng/ml. In concordance with our results, its expression was significantly increased in patients who experienced disease relapse (23). Ramnarine et al. (2018) revealed that several lncRNAs were involved in developing PCa (24). Zhang et al. (25) indicated that cell proliferation in hormone-refractory PCa was stimulated by lncRNAs (25). Similarly, our results demonstrated a significant increase in onco-lncRNAs, such as PCAT19, MALAT1, and NEAT1, and a decreased expression level of CASC2 in PCa patients.

Additionally, several studies investigated the interactions between lncRNAs, miRNAs, and mRNAs in various malignancies. A study indicated that elevated miRNA-182-5p was associated with cancer cells’ mitotic arrest through MALAT1 expression (26). Wang et al. (27) revealed an inverse correlation of expression between miRNA-195-5p and FGF in human cancer tissues (27). Regarding the ceRNA hypothesis in PCa, Xu et al. (28) revealed a ceRNA network of 63 prostate cancer-specific lncRNAs, 13 miRNAs, and 18 mRNAs. They also established a predictive model for overall survival using HOXB5, GPC2, PGA5, and AMBN (28). Besides, Jiang et al. (29) constructed a ceRNA network that included 23 lncRNAs, 6 miRNAs, and 2 mRNAs differentially expressed in PCa. However, only 3 lncRNAs were significantly associated with overall survival (29).

Basically, androgen/androgen receptor (AR) signaling is crucial for both normal prostate development and tumorigenesis which regulates a series of cancer biological processes including cell proliferation and metastasis (30, 31). Interestingly, several studies confirmed that AR-bound ceRNAs are required for AR interaction (32, 33). AR could directly target miRNAs and lncRNAs in PCa cells, which were recognized by microarray or RNA sequencing (34, 35). These studies strongly suggested that several ceRNAs containing specific sequences could bind to AR and other steroid receptors to stop their transcriptional activities (36). In this case, androgen deprivation therapy (ADT) is the standard management for patients with recurrences after primary treatment (37). Moreover, most primary cancers acquire ADT resistance and progress to castration-resistant PCa (38). Therefore, there is a critical need to clarify the molecular mechanisms contributing to the development of AR-dependent patients for emerging substitute therapeutic opportunities in advanced PCa. AR’s role and interaction with the network are poorly described. However, recent studies provide substantial evidence for this crosstalk in PCa. Several lncRNA-miRNA-mRNA networks are aberrantly expressed with significant contributions to PCa cell initiation and progression (39, 40). Application of their inhibitors can limit off-target or non-specific effects by avoiding targeting ceRNA complexes. Ostling et al. (2011) showed 78 ceRNA inhibitors to significantly modulate AR reporter activity (41). Some of them may activate or repress mRNA translations (42, 43). Further studies are required to disentangle these conflicting outcomes.

### 4.1 Clinical implications and limitations

Herein, we investigated commonly expressed lncRNA, miRNA, and target genes at different stages of prostate cancer. We hypothesized that a better understanding of the lncRNA-miRNA-mRNA network might develop novel therapeutic options for PCa patients. Finding therapeutic targets expressed at all prostate cancer stages has several advantages. However, there are some advantages and disadvantages. We used a large number of data from different sources to minimize off-target findings. Our patient-derived data showed network overlap. Cancer patients at any stage of the disease will benefit from their medicinal properties. While there is an association between disease stage and biomarker expression levels, these may be considered predictive biomarkers for treatment response. Since we demonstrated a significant difference in the expression levels of indicated lncRNAs and miRNAs in normal tissue and benign prostate hyperplasia, these biomarkers can act as a potential diagnostic biomarker in combination with PSA to increase diagnostic accuracy. Taken together, we introduced several promising diagnostic biomarkers. Further studies should identify this issue as a second-level test for patients. Some limitations merit consideration. First, due to the limited sample size, we could not evaluate the diagnostic accuracy of indicated lncRNAs and miRNAs as predictive tools. Therefore, future studies are warranted in this regard. Second, bioinformatic analysis predicted an interaction between lncRNAs and miRNAs. *In vitro* assessments directly evaluate the potential interactions between each lncRNA and its associated miRNA. Third, assessing the prognostic significance of the indicated lncRNAs and miRNAs regarding other clinical outcomes of patients and survival is warranted.

## 5 Conclusion

We have identified a common lncRNA-miRNA-mRNA network associated with prostate cancer, which may be clinically valuable as potential predictive biomarkers. These biomarkers might also serve as novel therapeutic targets or potential prognostic indicators for PCa patients.

## Data availability statement

The data analyzed in this study is subject to the following licenses/restrictions: The data supporting this study’s findings are available from the corresponding author, AA, upon reasonable request. Requests to access these datasets should be directed to AA, aalizadeh@sina.tums.ac.ir.

## Ethics statement

The studies involving human participants were reviewed and approved by Iran University of Medical Sciences (NO: IR.IUMS.REC.1399.1348). The patients/participants provided their written informed consent to participate in this study.

## Author contributions

GV: study concept and design and manuscript revision. SK: sample processing, data analysis, and manuscript preparation. AMA: study concept and design, data analysis, and manuscript revision. MY: sample collection. MM: manuscript preparation. TR and MaB: sample processing. MR: manuscript preparation. EE, MoB, and AK: sample collection. All authors contributed to the article and approved the submitted version.
