# Supplementary material for: A systematic method introduced a common lncRNA-miRNA-mRNA network in the different stages of prostate cancer
Source: Front Oncol. 2023 May 8;13:1142275. doi: 10.3389/fonc.2023.1142275 (PMC10215985; doi:10.3389/fonc.2023.1142275)
Supplement: Supplementary file 1 [file Table_1.docx]

**S1.** The list of primers for Real-Time PCR

| **Reverse primer** | **Forward primer** | **Indexes** |
| --- | --- | --- |
| ACCCAAGAGTTGATGTCCTTTCT | AGCCCAACGATGACTACTTACT | ALB |
| CATTGTGGGCAAGGTGCTATT | CTGCCGCTTTGCAGGTGTA | CD44 |
| ACTTTAGCTTCGGGTCAATGC | ATTCTCAACACTCCAAACTGTGC | CXCL12 |
| GATGTCAAACTCACTCATGGCT | TCAAGGCGCATGTGAACTCC | IL10 |
| GTCACCTGGTCAGTTAGCGT | AGTAACCTGCGGATTGGCTTC | ITGB3 |
| AATCGCCAGCCAATTCTCTTT | CTACGAGACCAAGTGCAATCC | BDNF |
| CATCATACTTCTTACGTACAGGCA | TGGGCATGCTCATTCTTCTT | MET |
| CACAGTGGTAGACTCAGGGGT | TGTTGCTGATCCTACTGGGC | PLG |
| GCAGGTAATCCCAAAAGCGAC | GTTGCTGGTCACATTCCTGG | APOE |
| GTGAGGATTTTGTCTCCTTCCAA | GGTGGTGTTGTCATGTGTTCT | MMP1 |
| GTGAAGGCAGGAGACCAGAG | TCAAGCAGGAAGGAAAGCAT | F2 |
| TTCCTGCTTCGTATTAACATGCT | ATGCACGCGGATCGAGTTT | ITGA6 |
| AATCGGCCAAACTCATCATGG | AGACATTCGATCCCTCTACAACT | ITGA5 |
| CACCGTCGTGTACTTGAAGGG | CACCAGCAAGGAGTGTGTGTT | FGF18 |
| AGACCACCAGAGAGCATATTTTG | ATGAGCTTCCTCGTCCAATTCA | FAP |
| AGGTCTTTGCGGATGTCCACGT | CACCATTGGCAATGAGCGGTTC | B-actin |
| CCAGTCTCAGGGTCCGAGGTATTC | CGTAGCAGCACAAGAAATATTGGC | miRNA-195 |
| CTGCCCCAGCACAGCC | CTCTGGCTCCGTGTCTTCAC | miRNA-149 |
| CCAGTGCAGGGTCCGAGGT | TGCGGTTTGGCAATGGTAGAAC | miRNA-182 |
| CGGGGGCCCTCGTCTTACCC | GGCTGGGGACCTGAGGCGAT | miRNA-200c |
| CGCGGATCCTCAAATAGCCATCCTAGACT | CCGGAATTCTTTCTGGGTAGCCTTTAGC | miRNA-491 |
| CCATGATCACGAAGGTGGTTT | ATGCAGTCGAGTTTCCCACAT | U6 |
| GGTCTGTGCTAGATCAAAGGCA | AAAGCAAGGTCTCCCCACAAG | MALAT1 |
| CAAGAAGATTCTTATCAGCT | TCAGAACAGGGAACCATTGG | PCAT19 |
| GCTCCACACAGTGTAGTCA | CACACAGGCATTAGACAGA | GASC2 |
| TCTCCTTGCCAAGCTTCCTTC | TGGCTAGCTCAGGGCTTCAG | NEAT1 |
